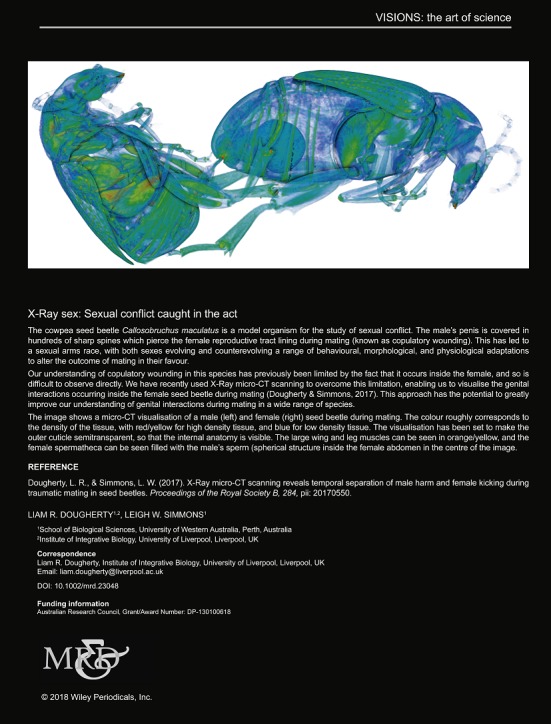# X‐Ray sex: Sexual conflict caught in the act

**DOI:** 10.1002/mrd.23046

**Published:** 2018-09-07

**Authors:** Liam R. Dougherty, Leigh W. Simmons

**Affiliations:** ^1^ School of Biological Sciences University of Western Australia Perth Australia; ^2^ Institute of Integrative Biology University of Liverpool Liverpool UK